# Leveraging football accelerometer data to quantify associations between repetitive head impacts and chronic traumatic encephalopathy in males

**DOI:** 10.1038/s41467-023-39183-0

**Published:** 2023-06-20

**Authors:** Daniel H. Daneshvar, Evan S. Nair, Zachary H. Baucom, Abigail Rasch, Bobak Abdolmohammadi, Madeline Uretsky, Nicole Saltiel, Arsal Shah, Johnny Jarnagin, Christine M. Baugh, Brett M. Martin, Joseph N. Palmisano, Jonathan D. Cherry, Victor E. Alvarez, Bertrand R. Huber, Jennifer Weuve, Christopher J. Nowinski, Robert C. Cantu, Ross D. Zafonte, Brigid Dwyer, John F. Crary, Lee E. Goldstein, Neil W. Kowall, Douglas I. Katz, Robert A. Stern, Yorghos Tripodis, Thor D. Stein, Michael D. McClean, Michael L. Alosco, Ann C. McKee, Jesse Mez

**Affiliations:** 1 Department of Physical Medicine and Rehabilitation, Harvard Medical School, Boston, MA USA; 2 Department of Physical Medicine and Rehabilitation, Massachusetts General Hospital, Boston, MA USA; 3 Department of Physical Medicine and Rehabilitation, Mass General Brigham-Spaulding Rehabilitation, Charlestown, MA USA; 4 Boston University Alzheimer’s Disease Research and CTE Centers, Boston University Chobanian & Avedisian School of Medicine, Boston, MA USA; 5 Department of Biostatistics, Boston University School of Public Health, Boston, MA USA; 6 Center for Bioethics and Humanities, University of Colorado Denver Anschutz Medical Campus, Aurora, CO USA; 7 Division of General Internal Medicine, University of Colorado School of Medicine, Aurora, CO USA; 8 VA Boston Healthcare System, U.S. Department of Veteran Affairs, Boston, MA USA; 9 Department of Veterans Affairs Medical Center, Bedford, MA USA; 10 Department of Pathology and Laboratory Medicine, Boston University Chobanian & Avedisian School of Medicine, Boston, MA USA; 11 Department of Epidemiology, Boston University School of Public Health, Boston, MA USA; 12 Concussion Legacy Foundation, Boston, MA USA; 13 Department of Neurosurgery, Boston University Chobanian & Avedisian School of Medicine, Boston, MA USA; 14 Department of Neurosurgery, Emerson Hospital, Concord, MA USA; 15 Department of Physical Medicine and Rehabilitation, Brigham and Women’s Hospital, Boston, MA USA; 16 Department of Neurology, Boston University Chobanian & Avedisian School of Medicine, Boston, MA USA; 17 Neuropathology Brain Bank & Research Core, Department of Pathology, Nash Family Department of Neuroscience, Ronald M. Loeb Center for Alzheimer’s Disease, Friedman Brain Institute, Icahn School of Medicine at Mount Sinai, New York, NY USA; 18 Department of Anatomy & Neurobiology, Boston University Chobanian & Avedisian School of Medicine, Boston, MA USA; 19 Framingham Heart Study, Boston University Chobanian & Avedisian School of Medicine, Boston, MA USA

**Keywords:** Neurodegenerative diseases, Neurodegeneration, Risk factors

## Abstract

Chronic traumatic encephalopathy (CTE) is a neurodegenerative tauopathy associated with repetitive head impacts (RHI), but the components of RHI exposure underlying this relationship are unclear. We create a position exposure matrix (PEM), composed of American football helmet sensor data, summarized from literature review by player position and level of play. Using this PEM, we estimate measures of lifetime RHI exposure for a separate cohort of 631 football playing brain donors. Separate models examine the relationship between CTE pathology and players’ concussion count, athletic positions, years of football, and PEM-derived measures, including estimated cumulative head impacts, linear accelerations, and rotational accelerations. Only duration of play and PEM-derived measures are significantly associated with CTE pathology. Models incorporating cumulative linear or rotational acceleration have better model fit and are better predictors of CTE pathology than duration of play or cumulative head impacts alone. These findings implicate cumulative head impact intensity in CTE pathogenesis.

## Abstract (web summary)

The relationship between the components of repetitive head impacts and chronic traumatic encephalopathy (CTE) remains unclear. Here, the authors use American football helmet sensor data to show that duration of play, cumulative head impacts and linear and rotational accelerations are significantly associated with CTE pathology.

## Introduction

Chronic traumatic encephalopathy (CTE) is a neurogenerative disease identified in individuals with exposure to repetitive head impacts (RHI), including military veterans, victims of physical violence, and contact and collision sports (CCS) athletes, including American football players^1,2^. CTE is distinguished from other neuropathologies by the pathognomonic lesion of perivascular accumulation of hyperphosphorylated tau in neurofibrillary tangles (NFTs), typically at the sulcal depths of the cerebral cortex, as well as diffuse NFTs in medial temporal lobe, diencephalon, basal ganglia, and brain stem^3^.

Effectively quantifying the RHI exposure needed to develop CTE has been limited and challenging. Importantly, self or informant reported symptomatic concussion has not been associated with the presence or severity of CTE pathology^1,4,5^. However, total years of football played, thought to serve as a proxy for RHI, demonstrated a significant dose-response relationship with both CTE presence and CTE severity^5^. The finding that years of play is positively associated with CTE pathology, whereas symptomatic concussion is not, suggests that other regularly occurring exposures, such as RHI, may have an instrumental role in influencing CTE development. However, the assessment of concussion is problematic, as concussion definitions and diagnoses have changed over time, and many athletes report vastly different numbers of concussions based on definitions applied^6^. This difficulty accurately quantifying concussions experienced is further compounded in studies that attempt to estimate concussion burden based on informant report.

American football provides a unique model to better understand the relationship between RHI and CTE. Athletes begin playing football at different ages and for different career durations. The average frequency of impacts that an athlete experiences over a given season, and the average intensity of those impacts, varies based on position and level played^7^. We hypothesized that these differences could be leveraged to investigate the relationships between RHI-related factors and CTE pathology, potentially with implications relevant for football players, as well as other contact sport athletes.

To estimate an athlete’s RHI exposure based on football position, duration, and level of play, data can be obtained from helmet accelerometers. Helmet accelerometers measure the head impact count and acceleration across different levels and positions of football play^8–41^. Building on the use of exposure matrices to quantify risks associated with exposures in other domains, data from helmet sensors have been previously utilized to approximate the potential number of head impacts that a football player may have received^42–45^. This cumulative head impact index (CHII), which incorporates an individual’s career length, positions, and levels of play, represents an athlete’s estimated lifetime number of football-related hits to the head^42–45^. CHII has been linked to former athletes’ mood and cognitive symptoms, cerebrospinal fluid total tau levels, and all-cause mortality, but the relationship to CHII and underlying pathology has not been previously studied^42–45^. Further, since the CHII was developed, more recent helmet sensor data from youth, high school, and collegiate football players have been published. These additional data (as well as older data) include estimates of average linear and rotational acceleration, measures of acceleration that were not included in the original CHII. Incorporation of these additional data may improve estimation of an athletes’ exposure to RHI and, by extension, improve estimation of the effect of RHI on CTE risk.

In this study, we sought to update the previously published CHII with updated data. Based on the well-established use of job exposure matrices to retrospectively characterize exposure to occupational hazards among workers^46^, we similarly developed a positional exposure matrix (PEM) to retrospectively characterize exposure to RHI among former football players. The PEM was derived from published helmet sensor studies that reported findings specific to position and level of play. We used this PEM to calculate CHIIs for deceased former football players whose brains were donated for neuropathological analysis. In addition, we expanded this PEM beyond number of impacts to include average acceleration of impacts, to allow for the calculation of estimated lifetime exposure to linear (CHII-G) and rotational (CHII-R) acceleration. We hypothesized that CHII, CHII-G, and CHII-R would be correlated with the presence and severity of CTE pathology, as well as NFT burden, and that models incorporating the intensity of impacts (CHII-G and CHII-R), would have better model fit and make better predictions of CTE presence, CTE severity, and NFT burden than models that do not incorporate these data (CHII and duration of play alone). We hypothesized that informant-reported concussion number and position would not be associated with CTE presence, CTE severity, or NFT burden.

## Results

### Brain donor characteristics

**Fig. 1:**
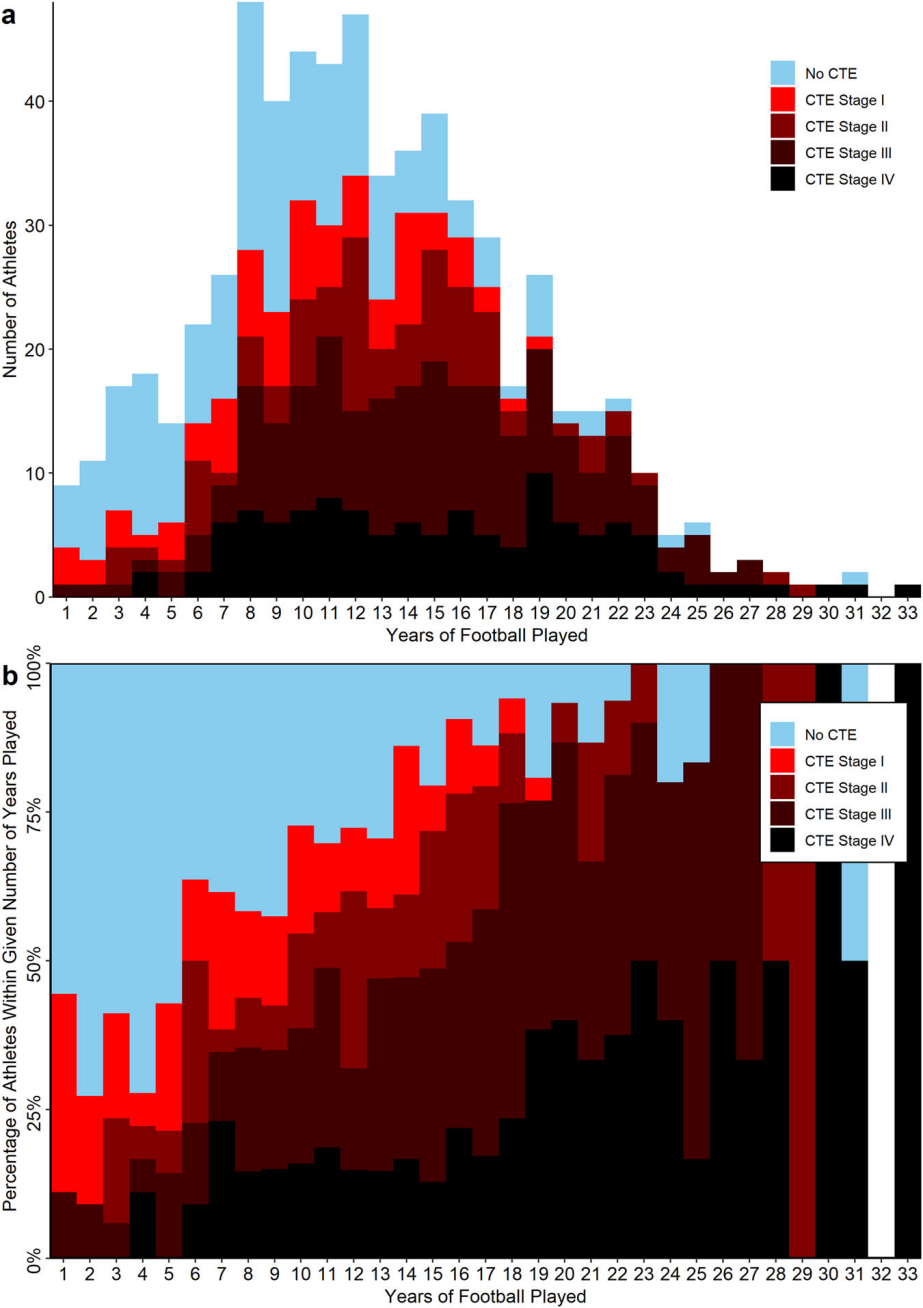
Athlete CTE Status by Years of American Football Played. Histograms (**a**) and percent distributions (**b**) for the study sample by duration of play. Of note: among the 69 athletes who played ≤5 years of football, 35 played another contact sport (13 of whom had CTE), 21 were in the military (7 of whom had CTE), and 9 were exposed to combat as part of military service (4 of whom had CTE). 47 of the 69 athletes had one or more of these exposures (16 of whom had CTE). Of the 69 athletes who played ≤5 years of football, 9 had CTE and none of these exposures. Please see Supplementary Fig. 1 for additional details. Source data are provided as a Source Data file. CTE chronic traumatic encephalopathy.

A total of 631 brain donors who played American tackle football were included in the study. Characteristics of all brain donors are presented in Tables 1–3. On average, athletes died at 59.7 years old (SD = 20.1) and played 12.5 years of football (SD = 5.9). 180 athletes did not have CTE, 163 had low-stage CTE (Stage I or II), and 288 had high-stage CTE (Stage III or IV). The absolute and relative distributions of athlete duration of play by CTE status/severity are presented in Fig. 1 (with distributions for exposure subgroups presented in Supplementary Fig. 1). The mean duration of play for those without CTE was 9.5 (SD = 5.3) years, for those with low-stage CTE was 11.6 (SD = 5.0) years and for those with high-stage CTE was 15.0 (SD = 5.7) years. The mean CHII, CHII-G and CHII-R for those without CTE was 4,515 hits (SD = 3,199), 87,489 total g-force (SD = 47,178) and 6.57 × 10^6^ total rad/s^2^ (SD = 4.35 × 10^6^) respectively; for those with low-stage CTE was 5,553 hits (SD = 3,410), 107,650 total g-force (SD = 41,755) and 8.32 × 10^6^ total rad/s^2^ (SD = 3.72 × 10^6^) respectively; and for those with high-stage CTE was 7,641 hits (SD = 3,870), 148,777 total g-force (SD = 52,557) and 12.26 × 10^6^ (SD = 5.0 × 10^6^) respectively. The three CHII measures were highly correlated (CHII vs. CHII-G: Pearson’s correlation (t) (degrees of freedom, df: 654) = 0.76, 95%CI: 0.72–0.79, *p* < 2.2 × 10^−16^; CHII vs. CHII-R: t(df: 654) = 0.66, 95%CI: 0.61–0.70, *p* < 2.2 × 10^−16^; CHII-G vs. CHII-R: t(df: 654) = 0.95, 95%CI: 0.95–0.96, *p* < 2.2 × 10^−16^).

**Table 1:** Demographic characteristics of brain donors by CTE status

	No CTE (*n* = 180)	Mild CTE (*n* = 163)	Severe CTE (*n* = 288)	All (*n* = 631)
Age at death, mean (SD)	53.0 (21.8)	47.5 (19.0)	70.9 (12.2)	59.7 (20.1)
Age at death, median (IQR; range)	57 (36,49; 13,97)	47 (30,62; 17,89)	73 (65,79; 30,97)	65 (46,76; 13,97)
Cause of death (%)				
Accidental overdose	12 (6.9%)	10 (6.3%)	5 (1.8%)	27 (4.4%)
Cancer	11 (6.3%)	10 (6.3%)	23 (8.2%)	44 (7.1%)
Cardiovascular disease	29 (16.6%)	28 (17.5%)	47 (16.7%)	104 (16.9%)
Injury	6 (3.4%)	9 (5.6%)	5 (1.8%)	20 (3.2%)
Motor neuron disease	2 (1.1%)	7 (4.4%)	12 (4.3%)	21 (3.4%)
Neurodegenerative disease	48 (27.4%)	14 (8.8%)	141 (50.0%)	203 (32.9%)
Suicide	46 (26.3%)	48 (30.0%)	14 (5.0%)	108 (17.5%)
Other	20 (11.4%)	34 (21.3%)	35 (12.4%)	89 (14.4%)
Unknown	1 (0.6%)	0 (0.0%)	0 (0.0%)	1 (0.2%)
Race (%)				
Asian	1 (0.6%)	0 (0.0%)	0 (0.0%)	1 (0.2%)
Black/African American	21 (11.7%)	29 (17.9%)	51 (17.7%)	101 (16.1%)
Native American/Alaskan	2 (1.1%)	1 (0.6%)	1 (0.3%)	4 (0.6%)
Pacific islander	1 (0.6%)	1 (0.6%)	1 (0.3%)	3 (0.5%)
White	151 (84.4%)	127 (78.4%)	234 (81.3%)	512 (81.4%)
Other	3 (1.7%)	4 (2.5%)	1 (0.3%)	8 (1.3%)
Sex (%)				
Female	0 (0.0%)	0 (0.0%)	0 (0.0%)	0 (0.0%)
Male	180 (100.0%)	163 (100.0%)	288 (100.0%)	631 (100.0%)
Education level (%)				
No high school	3 (1.7%)	0 (0.0%)	1 (0.3%)	4 (0.6%)
Some high school	10 (5.6%)	2 (1.2%)	0 (0.0%)	12 (1.9%)
High school/GED	21 (11.7%)	3 (1.9%)	4 (1.4%)	28 (4.4%)
Some college	47 (26.1%)	40 (24.7%)	46 (16.0%)	133 (21.3%)
College degree	56 (31.3%)	87 (53.7%)	150 (52.1%)	293 (46.5%)
More than college	6 (3.3%)	3 (1.9%)	22 (7.6%)	31 (4.9%)
Graduate degree	37 (20.6%)	27 (16.7%)	65 (22.6%)	129 (20.5%)

*CTE* chronic traumatic encephalopathy, *IQR* interquartile range, *SD* standard deviation

**Table 2:** Repetitive head impact exposure characteristics of brain donors by CTE status

	No CTE (*n* = 180)	Mild CTE (*n* = 163)	Severe CTE (*n* = 288)	All (*n* = 631)
Number of reported concussions, mean (SD)	67.0 (283.4)	89.2 (211.0)	115.1 (350.8)	94.5 (301.2)
Concussion number, median (IQR; range)	15 (6,50; 0,3500)	20 (7,87; 0,1500)	25 (9,100; 0,5000)	20 (7,92; 0,5000)
Age of first exposure to football (SD)	10.9 (3.8)	10.2 (3.6)	12.1 (2.8)	11.3 (3.4)
Duration of football in years (SD)	9.5 (5.3)	11.6 (5.0)	15.0 (5.7)	12.5 (5.9)
Highest level of football play (%)				
Pre-high school	10 (5.6%)	6 (3.7%)	1 (0.3%)	17 (2.7%)
High school	60 (33.3%)	29 (17.8%)	8 (2.8%)	97 (15.4%)
College	61 (33.9%)	64 (39.3%)	81 (28.1%)	206 (32.6%)
Semi-professional	16 (8.9%)	7 (4.3%)	4 (1.4%)	27 (4.3%)
Professional	33 (18.3%)	57 (35.0%)	194 (67.4%)	284 (45.0%)
Position played at highest level (%)				
Defensive back	10 (5.6%)	21 (12.9%)	29 (10.1%)	60 (9.5%)
Defensive line	23 (12.8%)	26 (16%)	37 (12.8%)	86 (13.6%)
Kicker	1 (0.6%)	1 (0.6%)	0 (0.0%)	0 (0.0%)
Linebacker	14 (7.8%)	26 (16.0%)	33 (11.5%)	73 (11.6%)
Offensive line	25 (13.9%)	23 (14.1%)	50 (17.4%)	98 (15.5%)
Punter	0 (0.0%)	2 (1.2%)	0 (0.0%)	2 (0.3%)
Quarterback	10 (5.6%)	7 (4.3%)	13 (4.5%)	30 (4.8%)
Running back	10 (5.6%)	10 (6.1%)	52 (18.1%)	72 (11.4%)
Tight end	8 (4.4%)	4 (2.5%)	13 (4.5%)	25 (4.0%)
Wide Receiver	5 (2.8%)	5 (3.1%)	10 (3.5%)	20 (3.2%)
Multiple	64 (35.6%)	32 (19.6%)	48 (16.7%)	144 (22.8%)
Other	4 (2.2%)	1 (0.6%)	0 (0.0%)	5 (0.8%)
Unknown	6 (3.3%)	5 (3.1%)	3 (0.1%)	14 (2.2%)
Participation in other contact/collision sport (%)	62 (34.4%)	53 (32.5%)	55 (19.1%)	170 (26.9%)
Military history (%)	41 (22.9%)	24 (14.8%)	82 (28.5%)	147 (23.4%)
Military combat exposure (%)	7 (3.9%)	7 (4.4%)	7 (2.4%)	21 (3.4%)
Exposure indices				
CHII, mean (SD)	4,515 (3,199)	5,553 (3,410)	7,641 (3,870),	6,117 (3,815)
CHII, median (IQR; range)	3963 (2191,6220; 206,18222)	4613 (3227,7027; 206,18384)	6841 (4830,9521; 206,19240)	5381 (3445,6118; 206,19240)
CHII-G, mean (SD)	88,972 (47,178)	107,650 (41,755)	148,777 (52,557)	119,081 (55,451)
CHII-G, median (IQR; range)	89,411 (60757,110939; 4246,280130)	110,054 (82076,131468; 4246,233872)	145,219 (106432,189016; 4246,310242)	112,741 (83737,151860; 4246,310242)
CHII-R, mean (SD)	6.57 × 10^6^ (4.35 × 10^6^)	8.32 × 10^6^ (3.72 × 10^6^)	12.26 × 10^6^ (5.0 × 10^6^)	9.48 × 10^6^ (5.19 × 10^6^)
CHII-R, median (IQR; range)	6.28 × 10^6^ (4.00 × 10^6^,8.30 × 10^6^; 0.25 × 10^6^,29.9 × 10^6^)	8.16 × 10^6^ (5.81 × 10^6^,10.4 × 10^6^; 0.25 × 10^6^,18.9 × 10^6^)	12.0 × 10^6^ (8.30 × 10^6^,15.5 × 10^6^; 0.25 × 10^6^.34.7 × 10^6^)	8.48 × 10^6^ (6.21 × 10^6^,12.69 × 10^6^; 0.25 × 10^6^,34.7 × 10^6^)

*CTE* chronic traumatic encephalopathy, *IQR* interquartile range, *SD* standard deviation

**Table 3:** Neuropathologic and clinical characteristics of brain donors by CTE status

	No CTE (*n* = 180)	Mild CTE (*n* = 163)	Severe CTE (*n* = 288)	All (*n* = 631)
Other neuropathologic diagnoses (%)				
Alzheimer’s disease	39 (21.7%)	4 (2.5%)	56 (19.4%)	99 (15.7%)
Frontotemporal lobar degeneration	11 (6.1%)	11 (6.8%)	26 (9.1%)	48 (7.6%)
Lewy body disease	24 (13.4%)	13 (8.0%)	64 (22.2%)	101 (16.1%)
Motor neuron disease	0 (0.0%)	7 (4.3%)	13 (4.5%)	20 (3.2%)
Diagnosed dementia (%)	63 (36.7%)	45 (28.0%)	216 (76.3%)	324 (52.6%)

*CTE* chronic traumatic encephalopathy, *IQR* interquartile range, *SD* standard deviation

### Informant-reported concussion number not associated with CTE status, CTE severity, or neurofibrillary tangle burden

Across all brain donors, there was no association between informant-reported concussion number and CTE status (odds ratio [OR] per concussion (df: 587) = 1.00, 95% confidence interval (CI) = 1.00–1.00; *p* = 0.23; Table 4). For athletes with CTE, there was no association between informant-reported concussion number and CTE severity (OR per concussion (df: 418) = 1.00, 95% confidence interval (CI) = 1.00–1.00; *p* = 0.49; Table 5). There was no association between informant-reported concussion number and cumulative neurofibrillary tangle (NFT) burden (mean difference in NFT burden index per concussion (df: 478) = 1.56 × 10^−4^, 95%CI = −1.96 × 10^−3^−2.28 × 10^−3^; *p* = 0.95; Table 6). All estimates above were adjusted for age at death (models unadjusted for age are included in Supplementary Tables 1–3).

**Table 4:** Association between exposure measures and CTE status (absent versus present)

	Odds ratio (95% CI)	*p*-value	BIC	Cross-validation mean error	AUC	*p*-value^a^
Concussion number	1.00 (1.00–1.00)	0.23				
Position, non-speed vs speed^b^	1.39 (0.89–2.16)	0.15				
Duration of play per year	1.15 (1.11–1.19)	9.4 × 10^−13^	685.3	0.177	0.716	
CHII per 1,000 hits	1.21 (1.13–1.29)	7.6 × 10^−9^	705.0	0.183	0.698	0.22
CHII-G per 10,000 g	1.20 (1.15–1.25)	2.0 × 10^−15^	665.9	0.170	0.757	9.5 × 10^−5^
CHII-R per 1,000,000 rad/sec^2^	1.22 (1.16–1.29)	7.0 × 10^−15^	665.2	0.169	0.763	1.5 × 10^−5^

Separate logistic regressions were run for each exposure measure due to multicollinearity to determine odds ratios, and *p*-values. For models with significant exposure measures, BIC and the mean error resulting from a 10-fold cross-validation analysis are reported to determine relative model performance. All models had the outcome of CTE status (absent vs present) and were adjusted for age at death

*AUC* receiver operating characteristics area under curve, *BIC* Bayesian information criterion, *CHII* cumulative head impact index representing estimated number of head impacts per donor per 1,000 hits, *CHII-G* cumulative head impact index representing estimated cumulative g-force experienced by each donor per 10,000 g, *CHII-R* cumulative head impact index estimated cumulative rotational force experienced by each donor per 1,000,000 rad/sec^2^, *CTE* chronic traumatic encephalopathy

^a^AUC *p*-value represents results of bootstrap analysis with 2000 replicates drawn from the sample to determine if there was a true difference between the AUCs for models examining CTE status and duration of play compared to other exposure measures

^b^Presented as dichotomous non-speed (offensive and defensive lineman) vs speed (all other positions) for all football players with a single known position

**Table 5:** Association between exposure measures and CTE severity (mild versus severe)

	Odds ratio (95% CI)	*p*-value	BIC	Cross-validation mean error	AUC	*p*-value^a^
Concussion number	1.00 (1.00–1.00)	0.49				
Position, non-speed vs speed^b^	1.37 (0.79–2.36)	0.26				
Duration of play per year	1.14 (1.08–1.20)	4.5 × 10^−7^	399.9	0.138	0.668	
CHII per 1,000 hits	1.13 (1.05–1.22)	9.2 × 10^−4^	418.2	0.144	0.674	0.75
CHII-G per 10,000 g	1.19 (1.12–1.26)	1.1 × 10^−8^	390.2	0.134	0.717	8.5 × 10^−7^
CHII-R per 1,000,000 rad/sec^2^	1.20 (1.13–1.28)	1.7 × 10^−8^	391.8	0.134	0.726	7.6 × 10^−6^

Separate logistic regressions were run for each exposure measure due to multicollinearity to determine odds ratios and *p*-values. For models with significant exposure measures, BIC and the mean error resulting from a 10-fold cross-validation analysis are reported to determine relative model performance. All models had the outcome of CTE severity (mild vs severe) and were adjusted for age at death

*AUC* receiver operating characteristics area under curve, *BIC* Bayesian information criterion, *CHII* cumulative head impact index representing estimated number of head impacts per donor per 1,000 hits, *CHII-G* cumulative head impact index representing estimated cumulative g-force experienced by each donor per 10,000 g, *CHII-R* cumulative head impact index estimated cumulative rotational force experienced by each donor per 1,000,000 rad/sec^2^, *CTE* chronic traumatic encephalopathy

^a^AUC *p*-value represents results of bootstrap analysis with 2000 replicates drawn from the sample to determine if there was a true difference between the AUCs for models examining CTE status and duration of play compared to other exposure measures

^b^Presented as dichotomous non-speed (offensive and defensive lineman) vs speed (all other positions) for all football players with a single known position

**Table 6:** Association between exposure measures and neurofibrillary tangle burden

	Mean increase in NFT burden per unit increase in respective measure (95% CI)	R^2^	*p*-value	BIC
Athletes with all 11 brain regions available for analysis (*n* = 519)				
Concussion number	1.56 × 10^−4^ (−1.96 × 10^−3^−2.28 × 10^−3^)	0.43	0.89	
Position, non-speed vs speed^a^	1.34 (−0.26–2.93)	0.35	0.10	
Duration of play per year	0.47 (0.36–0.57)	0.49	3.8 × 10^−16^	3558
CHII per 1,000 hits	0.44 (0.26–0.62)	0.45	1.8 × 10^−6^	3602
CHII-G per 10,000 g	0.51 (0.39–0.63)	0.49	2.6 × 10^−16^	3557
CHII-R per 1,000,000 rad/sec^2^	0.52 (0.39–0.65)	0.49	7.6 × 10^−15^	3564

Separate linear regressions were run for each exposure measure due to multicollinearity to determine betas, R^2^, and *p*-values. For models with significant exposure measures, BIC is reported to determine relative model performance. All models had the outcome of semi-quantitative NFT burden summed across 11 brain regions (0–33) and were adjusted for age at death. The sum score was based on neuropathologists semi-quantitative NFT burden on a 0–3 scale with increasing severity for 11 brain regions implicated in CTE: dorsolateral frontal cortex, middle frontal cortex, orbitofrontal cortex, hippocampus regions CA1, CA2, CA3/4, substantia nigra, amygdala, entorhinal cortex, inferior parietal cortex, and locus coeruleus. Results are presented for the 519 athletes with available tissue for all 11 brain regions

*BIC* Bayesian information criterion, *CHII* cumulative head impact index representing estimated number of head impacts per donor per 1,000 hits, *CHII-G* cumulative head impact index representing estimated cumulative g-force experienced by each donor per 10,000 g, *CHII-R* cumulative head impact index estimated cumulative rotational force experienced by each donor per 1,000,000 rad/sec^2^, *CTE* chronic traumatic encephalopathy, *NFT* neurofibrillary tangle

^a^Presented as dichotomous non-speed (offensive and defensive lineman) vs speed (all other positions) for all football players with a single known position

### Position at highest level not associated with CTE status, CTE severity, or neurofibrillary tangle burden

Across a range of analyses, primary playing position had no meaningful association with CTE related outcomes. We observed no association between football position played at the highest level and CTE status with positions evaluated individually (all ps > 0.05, Supplementary Table 4) or dichotomously (i.e., speed vs non-speed, defined previously^47^; OR(df; 467) = 1.39, 95%CI = 0.89–2.16; *p* = 0.15, Table 4). For those athletes with CTE (*n* = 362), there was no association between position played at the highest level and CTE severity (individually: all ps > 0.05; Supplementary Table 4. Dichotomously: *p* = 0.49; Table 6). There was no association between position played at the highest level and NFT burden (*n* = 385; *individually*: all ps > 0.05 except running back *p* = 0.002, with OR = 0.59, 95%CI = 4.4–801.8; Supplementary Table 4; *dichotomously*: *p* = 0.08; Table 6). These analyses restricted to brain donors with a single known position at the highest level of play. All models were adjusted for age at death.

### Duration and cumulative exposure measures associated with CTE status and severity

There was a significant association between duration of play, as well as the cumulative exposure measures, and CTE status (all ps < 0.001; Table 4), or severity (all ps < 0.001; Table 5), adjusted for age at death. Each additional year of play was associated with 15% increased odds of being diagnosed with CTE (df: 630, 95%CI: 1.11–1.19, *p* = 9.4 × 10^−13^) and, amongst those with CTE, 14% increased odds of being diagnosed with severe CTE (df: 450, 95% CI: 1.08–1.20, *p* = 4.5 × 10^−7^). Every additional estimated 1,000 head impacts was associated with 21% increased odds of being diagnosed with CTE (df: 630; 95%CI: 1.13–1.29, *p* = 7.6 × 10^−9^) and, among those with CTE, 13% increased odds of being diagnosed with severe CTE (df: 448; 95%CI: 1.05–1.22, *p* = 9.2 × 10^−4^). Every additional estimated 10,000 g cumulative linear acceleration to the head was associated with 20% increased odds of being diagnosed with CTE (df: 630; 95%CI: 1.15–1.25, *p* = 2.0 × 10^−15^) and, among those with CTE, 19% increased odds of being diagnosed with severe CTE (df: 450; 95%CI: 1.12–1.26, *p* = 1.1 × 10^−8^). Every additional estimated 1,000,000 rad/sec^2^ cumulative rotational acceleration to the head was associated with 22% increased odds of being diagnosed with CTE (df: 630; 95%CI: 1.16–1.29, *p* = 7.0 × 10^−15^) and, among those with CTE, 20% increased odds of being diagnosed with severe CTE (df: 450; 95%CI: 1.13–1.28, *p* = 1.7 × 10^−8^).

### CHII-G and CHII-R classified CTE status better than duration of play or CHII

Based on the Bayesian information criterion (BIC), there was very strong (∆BIC > 10) evidence for improved model fit for models estimating the relationship between CTE status and either duration of play, CHII-G, or CHII-R, compared with the model using CHII. Additionally, there was very strong evidence (∆BIC > 10) for improved model fit for models using either CHII-G and CHII-R over duration of play alone. There was no evidence of differences in model fit for the model incorporating CHII-G compared to CHII-R (∆BIC < 2).

**Fig. 2:**
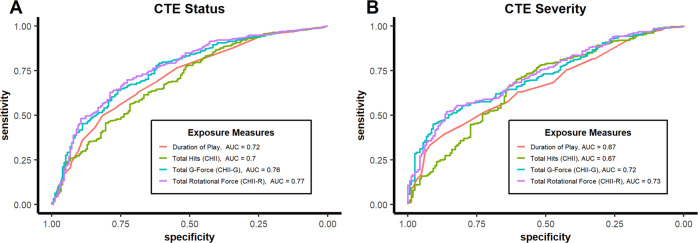
Performance of Exposure Measures as Classifiers of CTE Pathology. ROC Curves for Exposure Measures as Predictors of CTE Status (**A**) and CTE Severity (**B**). Source data are provided as a Source Data file. AUC area under the ROC curve, CHII cumulative head impact index representing estimated number of head impacts per donor, CHII-G cumulative head impact index representing estimated cumulative g-force experienced by each donor, CHII-R cumulative head impact index estimated cumulative rotational force experienced by each donor, CTE chronic traumatic encephalopathy, ROC receiver operating characteristics.

ROC analyses (Fig. 2A) indicated significant improvement in classifying CTE status for models using CHII-G (area under curve (AUC) =0.757, 95%CI: 0.715–0.798; *p* = 9.5 × 10^−5^) or CHII-R (AUC = 0.766, 95%CI: 0.725–0.807; *p* = 1.5 × 10^−5^), but not CHII (AUC = 0.698, 95%CI: 0.651–0.742; *p* = 0.22) compared to models using duration of play alone (AUC = 0.716, 95%CI: 0.675–0.760). There were similar significant improvements in classification using CHII-G (*p* = 2.6 × 10^−4^) and CHII-R (*p* = 9.9 × 10^−5^) compared to CHII. There were no significant differences between CHII-G and CHII-R in classifying CTE status (*p* = 0.19). Similarly, 10-fold cross validation analyses found lowest mean squared error (MSE) for CHII-R (MSE = 0.169) and CHII-G (MSE = 0.170), followed by duration of play (MSE = 0.177) and then CHII (MSE = 0.183).

### CHII-G and CHII-R better at classifying CTE severity than duration of play or CHII

When restricted to individuals with CTE, the BIC provided very strong evidence (∆BIC > 10) for improved model fit for models estimating the relationship between CTE severity and either duration of play, CHII-G, or CHII-R compared with the model using CHII. Additionally, there was strong evidence (∆BIC > 8) for improved model fit for models using either CHII-G and CHII-R over duration of play alone. There was no evidence of differences in model fit for the model incorporating CHII-G compared to CHII-R (∆BIC < 2).

ROC analyses (Fig. 2B) found significantly improved performance in classifying CTE severity for models using CHII-G (AUC = 0.717, 95%CI: 0.670–0.764; *p* = 8.5 × 10^−7^) or CHII-R (AUC = 0.726, 95%CI: 0.678–0.772; *p* = 7.6 × 10^−6^), but not CHII (AUC = 0.674, 95%CI: 0.620–0.725; *p* = 0.75) compared to models using duration of play alone (AUC = 0.668, 95%CI: 0.617–0.718). Similarly, there were significant improvements in classification using CHII-G (*p* = 0.014) and CHII-R (*p* = 0.009) compared to CHII. There were no significant differences between CHII-G and CHII-R in classifying CTE status (*p* = 0.21). Similarly, 10-fold cross validation analyses found lowest mean error for CHII-R (MSE = 0.134) and CHII-G (MSE = 0.134), followed by duration of play (MSE = 0.138) and then CHII (MSE = 0.144).

### Duration of play, CHII-G, or CHII-R preferred over CHII for modeling NFT burden

In models adjusted for age at death, there was also a significant relationship between increasing NFT burden and increasing years of football played, in addition to all cumulative exposure measures (*n* = 519, all ps < 0.001, Table 6). The models incorporating duration of play, CHII-G, and CHII-R, had higher R^2^s and showed very strong evidence for improved model fit (∆BIC > 10, Table 6) compared to models incorporating CHII.

*Sensitivity analyses: 1) after removing athletes who played multiple contact sports, 2) after removing athletes who had other neurodegenerative pathologies, and 3) without adjustment for age at death*.

While all athletes included in the above analyses played football, many had other exposures to RHI besides football; 26.9% of athletes (*n* = 170) participated in contact sports besides football and 23.4% (*n* = 147) served in the military (Supplementary Fig. 1). Similar relationships as reported above were observed between exposure measures and CTE outcomes when restricted to athletes with no military history or other contact sport exposure besides football (Supplementary Tables 5 and 6).

Additionally, some athletes had other neurodegenerative diseases besides CTE; 15.7% of athletes had Alzheimer’s disease (*n* = 99), 7.6% had frontotemporal lobar degeneration (*n* = 48), 16.1% had Lewy body disease (*n* = 101), and 3.2% had motor neuron disease (*n* = 20). Similar relationships as reported above were also observed when excluding athletes with any other neurodegenerative process (Supplementary Tables 5 and 6).

Adjusting for age at death may remove some of the variance in the neuropathological outcomes that the RHI exposures measures may explain. To investigate if this was the case, we re-ran our analyses without age in the models. Point estimates for the RHI exposure measures did not meaningfully change, but overall model fit and predictive power were mildly reduced (Supplementary Tables 1–3).

## Discussion

We investigated which components of RHI exposure from football play may be implicated in CTE pathology. The PEM reported here adapts the use of exposure matrices from other disciplines, and builds upon the previous use of CHII to quantify exposure to RHI. To our knowledge, this is the first study to use a PEM to estimate the relationship between different forms of RHI exposure and CTE pathology.

This PEM is a tool for quantifying a football player’s lifetime exposure to RHI, by estimating the cumulative number of head impacts as well as the cumulative linear and rotational accelerations associated with those impacts. We demonstrated an association between cumulative RHI exposure and CTE status, CTE severity, and NFT burden in football players. In general, model performance and fit using cumulative linear and rotational accelerations were improved compared to models with cumulative number of head impacts and years of play. Additionally, we found no relationship between informant-reported concussion number and CTE status, CTE severity, or NFT burden. Position played at the highest level was not associated with CTE status, CTE severity, or NFT burden, with the exception of a relationship observed between NFT burden, but not CTE status or severity, for running backs compared to offensive linemen.

These results provide additional evidence that repeated nonconcussive injuries are associated with CTE pathology. This is in contrast to the emphasis on concussions that is often discussed in the medical and lay literature^48^. Further, these results suggest that models incorporating intensity of impacts (i.e., linear and rotational acceleration) have better model fit and are better at predicting CTE status and severity than models incorporating duration of play or number of hits to the head alone. These results, if validated, could be used to identify changes to policy or gameplay that might limit CTE risk by decreasing cumulative exposure, such as by limiting duration of exposure, the number of exposures, and the magnitude of those exposures.

This study also validates the use of a PEM to characterize the aspects of RHI exposure experienced by football players. Other outcomes besides CTE believed to be associated with RHI exposure, such as measures of diffuse axonal injury^49^, neuroinflammatory markers^50,51^, or other neuropathologic processes^52^, could be evaluated in the context of exposures derived from the PEM to better understand the characteristics of the exposure most responsible for these changes.

Several biomechanical factors may explain why models testing the association between cumulative acceleration and CTE pathology have better fit than models incorporating the number of head impacts alone. Increasing linear forces are associated with focal injuries^53^; greater forces may therefore result in more areas of localized damage^54^. Furthermore, rotational forces may result in increased shear-induced damage, particularly around small blood vessels and at the depths of the sulci^49^.

This study utilized a PEM generated solely from available helmet accelerometer literature. Helmet accelerometer studies typically report results pertaining to head impacts greater than 10–15 g^35^. These thresholds were chosen to exclude incidental movements and jostling between the helmet and head^55^, but there is no evidence that these thresholds are most relevant to CTE pathology^56^. Future work should explore different thresholds for counting hits, to further characterize the nature of head impacts most responsible for CTE pathology.

The position an athlete plays serves as a proxy for the RHI exposure experienced. The fact that position at the highest level was not associated with CTE status, CTE severity, or NFT burden (the latter for all positions but running backs), whereas cumulative measures incorporating position were, suggests that one single position inadequately represents an athlete’s lifetime RHI exposure. Cumulative exposure measures appear to better approximate RHI exposure compared to single positions, likely because the former better accounts for the fact that football players change positions throughout their careers and often play multiple positions. Future measures should incorporate special teams participation (e.g. kickoff and punt), as this can vary by week and RHI from these positions may not be otherwise incorporated.

This study has several limitations. The study consists of a convenience sample of football playing brain donors, who tend to have greater exposure to RHI than the general population of football players. Even the athletes with lower years of exposure to football often had exposure to RHI from other sources, including other contact sports or military service, which were not characterized in this study. Along these lines, 82% of the subjects in this study played at the college level or above; predictions and thresholds from the present study would likely be most applicable to athletes with similar high levels of exposure. However, a substantial number of subjects had lower exposures (*n* = 17 with only youth participation and *n* = 95 only through high school), so we are not extrapolating to exposure ranges for which we have no data.

There are several sources of potential measurement error related to the cumulative RHI exposure measures: These measures were not observed directly while the donors were alive, but instead extrapolated from helmet accelerometer studies of other recent football players. Additionally, play style has changed over decades^57^, and many of the donors played decades ago, but the extrapolated data do not reflect these changes. Some studies reported means while others reported medians and we considered them equivalently. Also, the helmet accelerometer technology and the minimum thresholds for measured hits (e.g., 10 g vs 15 g) differed across studies. These studies also reported averages across all athletes at a given level, but donors in this cohort, particularly those that ultimately played professionally, likely had different RHI exposures than an average athlete. Additionally, accelerometers in helmets may move independently from the head and may not accurately indicate acceleration experienced by the brain^58^. Because the professional football leagues have not made their helmet sensor data available, we extrapolated collegiate athlete exposure to professional athletes. Furthermore, athletes play multiple positions even within a given season; these analyses averaged up to two positions per season but may not accurately reflect uneven distributions of playing time for a given athlete. Special teams participation could introduce measurement error, as impacts during kickoffs and punts, which tend to be high magnitude, were not incorporated into the PEM-derived cumulative exposure measures. Even with several sources of potential measurement error, measurement error can be overcome with a sufficiently large sample size, provided there is not bias in the estimation. We do not have reason to suspect that measurement error differed by neuropathological status to introduce bias. Given that we were able to find a robust relationship between the estimated cumulative measures of RHI exposure and CTE pathology in the absence of a reasonable explanation for bias, it seems this sample size was sufficiently large to overcome potential measurement error. There are additional limitations. CTE diagnoses and staging may have been obscured by comorbid pathology. However, we would not expect this to bias the reported results as the pathologists were blinded to the athletic history, upon which the RHI exposure measures were based. Concussion number was also obtained retrospectively from informant report and validated with medical records where available; given changes in concussion diagnoses over decades^57^, as well as recall bias and use of informant report, these reported concussion numbers may not accurately reflect concussion exposure. As a result, it is possible that a true relationship between number of symptomatic head impacts and CTE pathology exists, but that measurement error obscured this relationship. However, these difficulties in diagnosing concussions are neither new, nor resolved, so the present study demonstrates a potential means of estimating head impact burden that is robust to these issues.

This work utilized a PEM to better characterize which components of RHI exposure from football play may be implicated in CTE pathology. Models incorporating impact intensity, in addition to duration of exposure and number of impacts, improved model fit and prediction of CTE pathology compared with models without impact intensity. These findings provide insight into the pathogenesis of CTE, implicate potential targets for CTE interventions, and indicate that future studies may benefit from using PEM-derived measures of RHI exposure.

## Methods

### Brain donor recruitment

**Fig. 3:**
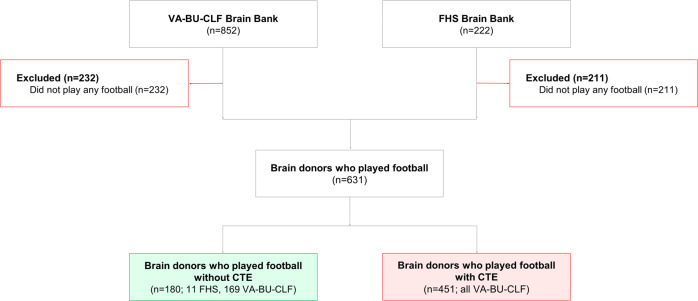
Study inclusion and exclusion criteria for FHS and VA-BU-CLF study brain donors. Source data are provided as a Source Data file. BU Boston University, CLF Concussion Legacy Foundation, FHS Framingham Heart Study, VA Veterans Affairs.

All participants were former football players from the Understanding Neurologic Injury and Traumatic Encephalopathy (UNITE) and Framingham Heart Study (FHS) Brain Banks (Fig. 3), which have been previously described^5^. Donors to the UNITE Brain Bank must have had a history of repetitive head impact (RHI) exposure through contact and collision sports (CCS), military service, or domestic violence. Recruitment to the UNITE Brain Bank began in 2008 and is ongoing; the present study included donors through 2020. Since tracking began in 2014, recruitment occurred in the following ways. Next-of-kin contacting the brain bank near or at the time of death was the most common recruitment method (*n* = 433). Other forms of recruitment included individuals joining the CLF Brain Donation Registry during life (*n* = 18), a medical examiner contacting the brain bank (*n* = 33), a CLF representative contacting the next-of-kin after the time of death (*n* = 19), and a consult request (*n* = 1). No sex or gender analyses were carried out as all football players in this cohort were male.

FHS is a prospective surveillance study tracking incident cardiovascular disease, stroke, and dementia. The study was established in 1948, enrolling a representative group from Framingham, Massachusetts and subsequently their children (Gen 2) and grandchildren (Gen 3), as well as ethnically diverse cohorts (Omni 1&2) to better capture changing demographics of Framingham. The FHS Brain Bank contains a voluntary subset of these cohorts, and began recruiting in 1997. Because the FHS is a community-based sample, players tended to be older and to have fewer years of American football play. Their addition resulted in more total players without CTE and in a similar distribution of age-at-death between players with and without CTE.

For both brain banks, prospective donors were excluded if there was a postmortem interval for donation greater than 72 hours, or if fragments or less than a hemisphere of tissue were received. Consent from participant’s next-of-kin was required to participate. Institutional review board approval was obtained through Boston University Medical Campus and Bedford VA Hospital.

### Clinical data

For the UNITE Brain Bank, previously detailed methods for retrospective clinical data collection and comprehensive review of all relevant medical records were followed for all participants. A structured clinical history was obtained from informants, including a timeline of cognitive, behavioral, mood and motor symptomatology. Clinicians with expertise in neurodegenerative disease reviewed all cases to reach consensus on a dementia diagnosis based on DSM-IV-TR criteria. Clinicians and clinical research assistants were blinded to the neuropathological examination and findings^59^.

For the FHS brain bank, clinical data were obtained prospectively as part of the FHS clinical assessment. Participants underwent cognitive assessment and those with suspected cognitive impairment were brought to a consensus meeting, during which it was determined whether the donor met criteria for dementia^60^.

### Contact sport and traumatic brain injury (TBI) history

Retrospective assessment of contact sport and TBI histories from informants were similar in both brain banks and have been described previously^1,5,59^. For each CCS sport exposure (football or otherwise), informants provided a history of levels played, years played at each level, and age of first exposure. Football position at each level was also recorded. In addition, athletic history for professional football players was verified with a comprehensive database maintained by Hidden Game Sports/24-7 Baseball LLC, which has been previously used for research purposes^1,5,61^. Informants were also asked if donors had any military service or combat exposure. Each donor’s TBI history was queried from informants, including assessing the total number of concussions experienced. Following amendments made to study protocols, informants for donors included in the study after January 2014 were read a formal definition of concussion in advance of being asked how many concussions each donor experienced^6^.

### Neuropathological examination

Methods for brain tissue processing and evaluation have been described previously^1,4^. Neuropathologists were blinded to the donor’s exposure history, all clinical history, and medical diagnoses. All pathologic diagnoses were reviewed by four neuropathologists (A.C.M., B.R.H., T.D.S., V.E.A.) and discrepancies were resolved by discussion. Gross and microscopic examination were conducted following previously published methods^62^. Well-established criteria were used for the neuropathologic diagnosis of neurodegenerative diseases, including Alzheimer’s disease (NIA-Reagan criteria of high or intermediate likelihood)^63^, Lewy body disease^64^, frontotemporal lobar degeneration^65,66^, and motor neuron disease^67^. CTE was diagnosed using NINDS/NIBIB neuropathological criteria^3^. Donors with CTE were staged (I to IV, in order of increasing severity) based on previously published criteria^4,68^, which were later classified as low-stage CTE (stages I and II) and high-stage CTE (stages III and IV). Neuropathologists also recorded semi-quantitative NFT burden on a 0–3 scale with increasing severity across 11 brain regions implicated in CTE: dorsolateral frontal cortex, middle frontal cortex, orbitofrontal cortex, hippocampus regions CA1, CA2, CA3/4, substantia nigra, amygdala, entorhinal cortex, inferior parietal cortex, and locus coeruleus. Cumulative neurofibrillary tangle (NFT) burden was defined as the sum score across these 11 regions.

### Helmet sensor study literature review and creation of the positional exposure matrix (PEM)

We created a PEM that quantified RHI features specific to football position and level of play, adapting the template of the job exposure matrix, which is used in the field of occupational health to retrospectively characterize exposure^46^. This involved three steps: (1) identifying published helmet accelerometer studies reporting hits per season, linear acceleration, and rotational acceleration specific to position and level of play; (2) abstracting measures of central tendency (e.g., mean or median hits per season) from each report; and (3) computing summary means of hits per season, linear acceleration, and rotational acceleration, specific to position and level of play, with each study’s estimate weighted in proportion to its sample size.

First, data were compiled from previously published helmet accelerometer studies in football players that reported either the number of impacts sustained per season, average linear acceleration sustained each season, and/or average rotational acceleration sustained each season^8–41^. Specifically, a literature review was conducted using PUBMED to identify articles published prior to 2021 with the search terms: “head impact telemetry system,” “football helmet accelerometer,” “football helmet linear acceleration,” and “football helmet rotational acceleration”. These articles were reviewed and included in the positional exposure matrix (PEM) if they fit the following criteria:

- Head impacts were measured across *practices and games for the entire season*
- *Level of play* (youth, high-school, college) was identified
- *Mean or median* head impact frequencies, linear acceleration, or rotational acceleration were reported by *position played* (only for high-school and beyond, given that no studies reported results for youth by position played)
- Any impact event with a peak linear acceleration <10 *g* was excluded from analysis. A minimum *cutoff of 10* g *ensures* the elimination of nonimpact events (e.g., jumping) from the calculation of head impact frequency

Based on these criteria, 34 articles were identified.

We next compiled values that were either directly reported in a specific paper or that we derived from the data reported (e.g., a paper might have reported total participants and cumulative hits across all participants; mean frequency of hits per season was derived by dividing cumulative hits per season by number of participants). Values were derived using arithmetic if not directly reported. The contributing studies reported either the mean or median values, both were included in the PEM and treated similarly. When studies grouped multiple positions together in their results (e.g., simply reporting results for “speed” and “non-speed” positions), the aggregate information provided for each group was applied to all positions within that group, for that study. Cumulative head impacts, linear acceleration, and rotational acceleration for a single player across a single season were reported and derived if necessary. Mean or median impact frequencies, linear acceleration, and rotational acceleration across a single season were weighted by each study’s sample size. These weighted averages are the impacts experienced per position per season at the different levels of play (youth, high school, college).

Finally, these data were used to develop the PEM. The PEM aggregated the weighted mean annual numbers and intensities (linear and rotational acceleration) of exposures to head impacts across all reported positions and levels of play. For any missing information in the PEM based on lack of available helmet sensor data (e.g., no reports of the rotational acceleration experienced by collegiate defensive backs), the average data for that level across all positions was used (e.g., the mean rotational acceleration across all collegiate positions weighted by study sample size). Because there is currently no helmet sensor data for semi-professional or professional football players, collegiate data from the PEM were used to approximate these levels of play. Table 7 provides a summary of values obtained from key variables in the PEM, with the corresponding data reported in Supplementary Source Data, per PRISMA guidelines^69^.

**Table 7:** Position exposure matrix of weighted average annual exposures aggregated from previously published helmet sensor studies

	Number of hits per season			Linear acceleration (g)			Rotational acceleration (rad/s^2^)		
	Youth	High school	College	Youth	High school	College	Youth	High school	College
**Overall**	206.4	538.7	526.0	20.6	26.5	20.6	1203.4	1898.4	1574.2
**DL**		782.3	840.9		25.8	21.0		1801.3	1806
**DB**		316.6	371.6		28.5	20.2		1957.4	
**LB**		460.2	539.0		27.3	22.3		1870.4	2071.7
**OL**		734.4	814.6		25.8	21.0		1777.5	1782
**QB**		320.2	209.4		26.8	21		1476.4	
**RB**		475.1	421.7		27.7	21.8		1807.7	1878.8
**TE**		517.4	599.2		27.1	31.0		1625.8	1815.7
**WR**		301.9	313.9		28.8	19.5		2223.8	

Source data are provided as a Source Data file

*DL* defensive line, *DB* defensive back, *LB* linebacker, *OL* offensive line, *QB* quarterback, *RB* running back, *TE* tight end, *WR* wide receiver

### Cumulative head impact indices (CHIIs)

The PEM was used to calculate three indices of exposure to head impacts over each athlete’s lifetime. The indices, which correspond with the three head impact features in the PEM, were the estimated number of the impacts (CHII), the estimated total linear acceleration sustained (CHII-G), and the estimated total rotational acceleration sustained (CHII-R). For each athlete, CHII was calculated:

1 $${{{{{\rm{CHII}}}}}}=\mathop{\sum }\limits_{n=1}^{y}{h}_{n}$$

where *y* is the number of years the athlete played football and *h* is the number of head impacts an average athlete is exposed to annually according to the PEM, given the position and level that the athlete played, during year *n*. For example, an athlete who played one year of football in high school as a linebacker, one year of football in high school as a defensive lineman, and one year in college as a defensive lineman, would have (numbers from PEM in Table 7):

2 $${{{{{\rm{CHII}}}}}}	=\mathop{\sum }\limits_{n=1}^{y}{h}_{n}={h}_{1}+{h}_{2}+{h}_{3}\\ 	=(mean\,weighted\,annual\,frequency\,of\,high\,school\,linebacker\,hits)\\ 	+(mean\,weighted\,annual\,frequency\,of\,high\,school\,defensive\,lineman\,hits)\\ 	+(mean\,weighted\,annual\,frequency\,of\,college\,defensive\,lineman\,hits)\\ 	=460.2+782.3+840.9=2,083.4\,{{{{{\rm{estimated}}}}}}\,{{{{{\rm{lifetime}}}}}}\,{{{{{\rm{cumulative}}}}}}\,{{{{{\rm{hits}}}}}}$$

Similarly, CHII-G was calculated:

3 $${{{{{\rm{CHII}}}}}}-{{{{{\rm{G}}}}}}=\mathop{\sum }\limits_{n=1}^{y}{h}_{n}{g}_{n}$$

where *g* is the linear acceleration (measured in g-force) that an average athlete is exposed to annually according to the PEM, given the position and level that the athlete played, during year *n*. For the above example athlete:

4 $${{{{{\rm{CHII}}}}}}-{{{{{\rm{G}}}}}}	=\mathop{\sum }\limits_{n=1}^{y}{h}_{n}{g}_{n}={h}_{1}{g}_{1}+{h}_{2}{g}_{2}+{h}_{3}{g}_{3}\\ 	={h}_{1}\times (mean\,weighted\,annual\,g-force\,of\,high\,school\,linebacker\,hits)+{h}_{2}\\ 	 \times (mean\,weighted\,annual\,g-force\,of\,high\,school\,defensive\,lineman\,hits)+{h}_{3}\\ 	 \times (mean\,weighted\,annual\,g-force\,of\,college\,defensive\,lineman\,hits)\\ 	=460.2\times (27.3)+782.3\times (25.8)+\,840.9\times (21.0)\\ 	=50,405.7\,{{{{{\rm{estimated}}}}}}\,{{{{{\rm{lifetime}}}}}}\,{{{{{\rm{cumulative}}}}}}\,{{{{{\rm{g}}}}}}-{{{{{\rm{force}}}}}}$$

Similarly, CHII-R was calculated:

5 $${{{{{\rm{CHII}}}}}}-{{{{{\rm{R}}}}}}=\mathop{\sum }\limits_{n=1}^{y}{h}_{n}{r}_{n}$$

where *r* is the rotational acceleration (measured in rad/sec^2^) that an average athlete is exposed to annually, according to the PEM, given the position and level that the athlete played, during year *n*. For the above example athlete:

6 $${{{{{\rm{CHII}}}}}}-{{{{{\rm{R}}}}}}	=\mathop{\sum }\limits_{n=1}^{y}{h}_{n}{r}_{n}={h}_{1}{r}_{1}+{h}_{2}{r}_{2}+{h}_{3}{r}_{3}\\ 	={h}_{1}\times (mean\,weighted\,annual\,rad/sec2\,of\,high\,school\,linebacker\,hits)+{h}_{2}\\ 	 \times (mean\,weighted\,annual\,rad/sec2\,of\,high\,school\,defensive\,lineman\,hits)+{h}_{3}\\ 	 \times (mean\,weighted\,annual\,rad/sec2\,of\,college\,defensive\,lineman\,hits)\\ 	=460.2\times (1870.4)+782.3\times (1801.3)+840.9\times (1806)\\ 	=3,788,580.5\,{{{{{\rm{estimated}}}}}}\,{{{{{\rm{lifetime}}}}}}\,{{{{{\rm{cumulative}}}}}}\,{{{{{\rm{rotational}}}}}}\,{{{{{\rm{acceleration}}}}}}$$

For athletes who played multiple positions during a given year, the respective exposure index for that year was the mean exposure index of the multiple positions. Previous iterations of the CHII asked athletes to estimate the percent of time that they played at any positions they played within a given season. However, in pilot testing, when informants were asked to estimate the percentage of time that their loved ones spent playing at a given position, informants consistently reported that they that they did not know. As such, the questions asking percent time played at each level were not incorporated into the present study.

### Statistical methods

Separate logistic regression models were fitted to determine the association between each exposure measure (concussion number, position at highest level, duration of football play, CHII, CHII-G, and CHII-R) and CTE status (absent or present). Among those with CTE, we fitted parallel models using CTE severity (low or high) as the outcome. Because we sought information about individual-level prediction, receiver operating characteristics (ROC) curves were plotted for all significant exposure measures (*p* < 0.05) to observe the relationship between each exposure measure and both dichotomous CTE outcomes. The bootstrap approach was used to evaluate differences in discriminative power between models^70^. To further evaluate predictive ability of each model, a 10-fold cross-validation study was performed for each significant exposure measure and both dichotomous CTE outcomes.

We fitted linear regressions to quantify the association of each exposure measure with NFT burden, a semi quantitative measure consisting of a zero to three score for each region, summed across 11 brain regions (total range: 0–33). These analyses were restricted to data from athletes with all 11 brain regions available for neuropathological evaluation (82.1%). Imputation was not performed for missing brain region data because we were sufficiently powered without imputation. Additionally, missingness was not associated with exposure measures after adjusting for age at death, so estimated effects were unlikely to be biased by differential missingness.

All models were adjusted for age at death given its known association with CTE pathology. For all models, Bayesian information criterion (BIC) was calculated to aid in comparison of model fits. To adjust for multiple analyses, the Bonferroni-adjusted significance level of 0.0083 was used. All data were collected and secured using Boston University Medical Center REDCap. All analyses were performed with R v4.0.5 or SPSS v.27.0.1.0. Given the high-profile and sensitive information for these donors, access to data is strictly monitored to ensure confidentiality. De-identified biospecimens and limited dataset clinical are available upon submission of IRB approved proposal and data use agreement. Please contact corresponding author to initiate this process. All code is available upon request to the corresponding author.

### Reporting summary

Further information on research design is available in the Nature Portfolio Reporting Summary linked to this article.

## Supplementary information

Supplementary Information

Description of Additional Supplementary Files

Supplementary Code

Reporting Summary

## Data Availability

The raw datasets generated during and/or analyzed during the current study are not publicly available due to potential ability to identify elite athletes based on exposure data. However, these data are available from the corresponding author on request and with relevant IRB approval. Source data are provided with this paper.

## Source data

Source Data

## Code availability

All code used for the present project, as well as a sample dataset, has been provided and is available as the supplementary code file.
