# Supplementary material for: Leveraging football accelerometer data to quantify associations between repetitive head impacts and chronic traumatic encephalopathy in males
Source: Nat Commun. 2023 Jun 20;14:3470. doi: 10.1038/s41467-023-39183-0 (PMC10281995; doi:10.1038/s41467-023-39183-0)
Supplement: Supplementary file 1 — Supplementary Information [file 41467_2023_39183_MOESM1_ESM.pdf]

Supplementary Tables and Figures

Supplementary Table 1. Association between exposure measures and CTE status (absent versus present) in models unadjusted for age

	Odds ratio (95% CI)	p-value	BIC	Cross-validation mean error
Concussion number	1.00 (1.00-1.00)	.18		
Position, non-speed vs speed*	1.37 (0.89-2.13)	.15		
Duration of play per year	1.16 (1.12-1.20)	$5.4*10^{-15}$	692.3	0.181
CHII per 1,000 hits	1.24 (1.16-1.32)	$2.1*10^{-11}$	708.9	0.185
CHII-G per 10,000g	1.21 (1.16-1.27)	$<2.0*10^{-16}$	664.1	0.172
CHII-R per 1,000,000 rad/sec^2	1.25 (1.19-1.31)	$<2.0*10^{-16}$	662.4	0.168

Separate logistic regressions were run for each exposure measure due to multicollinearity to determine odds ratios, and p-values. For models with significant exposure measures, BIC and the mean error resulting from a 10-fold cross-validation analysis are reported to determine relative model performance. All models had the outcome of CTE status (absent vs present) and were adjusted for age at death.

*Presented as dichotomous non-speed (offensive and defensive lineman) vs speed (all other positions) for all football players with a single known position

BIC: Bayesian information criterion; CHII: cumulative head impact index representing estimated number of head impacts per donor per 1,000 hits; CHII-G: cumulative head impact index representing estimated cumulative g-force experienced by each donor per 10,000g; CHII-R: cumulative head impact index estimated cumulative rotational force experienced by each donor per 1,000,000 rad/sec^2; CTE: chronic traumatic encephalopathy

Supplementary Table 2. Association between exposure measures and CTE severity (mild versus severe) in models unadjusted for age

	Odds ratio (95% CI)	p-value	BIC	Cross-validation mean error
Concussion number	1.00 (1.00-1.00)	.44		
Position, non-speed vs speed*	1.11 (0.71-1.74)	.64		
Duration of play per year	1.13 (1.08-1.17)	$4.9*10^{-9}$	563.0	0.214
CHII per 1,000 hits	1.18 (1.11-1.26)	$1.0*10^{-7}$	532.2	0.215
CHII-G per 10,000g	1.20 (1.14-1.26)	$1.9*10^{-13}$	568.4	0.201
CHII-R per 1,000,000 rad/sec^2	1.23 (1.16-1.29)	$8.2*10^{-14}$	528.0	0.198

Separate logistic regressions were run for each exposure measure due to multicollinearity to determine odds ratios and p-values. For models with significant exposure measures, BIC and the mean error resulting from a 10-fold cross-validation analysis are reported to determine relative model performance. All models had the outcome of CTE severity (mild vs severe) and were adjusted for age at death.

*Presented as dichotomous non-speed (offensive and defensive lineman) vs speed (all other positions) for all football players with a single known position

BIC: Bayesian information criterion; CHII: cumulative head impact index representing estimated number of head impacts per donor per 1,000 hits; CHII-G: cumulative head impact index representing estimated cumulative g-force experienced by each donor per 10,000g; CHII-R: cumulative head impact index estimated cumulative rotational force experienced by each donor per 1,000,000 rad/sec^2; CTE: chronic traumatic encephalopathy

Supplementary Table 3. Association between exposure measures and neurofibrillary tangle burden in models unadjusted for age

	Mean increase in NFT burden per unit increase in respective measure (95% CI)	R^2	p-value	BIC
Athletes with all 11 brain regions available for analysis (n=519)				
Concussion number	$3.35*10^{-4}$ ($-2.46*10^{-3}$-$3.12*10^{-3}$)	0.00	.81	
Position, non-speed vs speed*	1.88 (-0.09-3.85)	0.01	.06	
Duration of play per year	0.67 (0.54-0.81)	0.15	$<2*10^{-16}$	3558
CHII per 1,000 hits	0.87 (0.65-1.09)	0.11	$2.1*10^{-14}$	3602
CHII-G per 10,000g	0.85 (0.71-0.98)	0.22	$<2*10^{-16}$	3557
CHII-R per 1,000,000 rad/sec^2	0.89 (0.74-1.04)	0.21	$<2*10^{-16}$	3564

Separate linear regressions were run for each exposure measure due to multicollinearity to determine betas, R^2, and p-values. For models with significant exposure measures, BIC is reported to determine relative model performance. All models had the outcome of semi-quantitative NFT burden summed across 11 brain regions (0-33) and were adjusted for age at death. The sum score was based on neuropathologists semi-quantitative NFT burden on a 0-3 scale with increasing severity for 11 brain regions implicated in CTE: dorsolateral frontal cortex, middle frontal cortex, orbitofrontal cortex, hippocampus regions CA1, CA2, CA3/4, substantia nigra, amygdala, entorhinal cortex, inferior parietal cortex, and locus coeruleus. Results are presented for the 519 athletes with available tissue for all 11 brain regions.

*Presented as dichotomous non-speed (offensive and defensive lineman) vs speed (all other positions) for all football players with a single known position

BIC: Bayesian information criterion; CHII: cumulative head impact index representing estimated number of head impacts per donor per 1,000 hits; CHII-G: cumulative head impact index representing estimated cumulative g-force experienced by each donor per 10,000g; CHII-R: cumulative head impact index estimated cumulative rotational force experienced by each donor per 1,000,000 rad/sec^2; CTE: chronic traumatic encephalopathy; NFT: neurofibrillary tangle

Supplementary Table 4. Association between position and CTE status, CTE severity, and NFT burden

	CTE status*		CTE severity**		NFT burden***	
Highest Position	Odds ratio (95% CI)	p-value	Odds ratio (95% CI)	p-value	Odds ratio (95% CI)	p-value
Defensive back	1.9 (0.85-4.6)	.12	0.96 (0.37-2.5)	.93	1.0 ($6.7*10^{-2}$-15.0)	1.00
Defensive line	0.91 (0.47-1.8)	.80	0.55 (0.23-1.3)	.17	0.61 ($5.3*10^{-2}$-7.0)	.69
Kicker	0.47 ($1.8*10^{-2}$-12.3)	.60	$6.9*10^{-7}$ (NA-$1.3*10^{-122}$)	.99	$2.8*10^{-4}$ ($1.7*10^{-6}$-1.9)	.29
Linebacker	1.6 (0.74-3.5)	.21	0.66 (0.27-1.6)	.37	1.3 (0.10-17.7)	.82
Punter	$7.2*10^{-5}$ ($2.2*10^{-5}$-NA)	.98	$9.4*10^{-9}$ (NA-$4.9*10^{-62}$)	.98	$1.6*10^{-3}$ ($3.3*10^{-8}$-76.0)	.24
Quarterback	0.61 (0.25-1.6)	.29	0.76 (0.21-3.1)	.70	4.9 (0.14-173.9)	.38
Running back	1.9 (0.84-4.4)	.14	2.4 (0.88-6.7)	.10	0.59 (4.4-801.8)	.002
Tight end	0.65 (0.25-1.8)	.39	1.2 (0.32-5.5)	.77	0.57 ($1.5*10^{-2}$-21.7)	.76
Wide Receiver	1.0 (0.35-3.5)	.96	1.4 (0.30-7.1)	.68	0.56 ($8.1*10^{-3}$-39.1)	.79
(Offensive line used as reference category)						

Each column represents the results of a logistic (CTE status and severity) or linear (NFT burden) regression with age at death as a covariate and highest position played as a factor variable (offensive line as the reference category).
*For all athletes with a single known highest position played (n=468)
**For all athletes with a single known highest position played and CTE (n=362)
***Athletes with a single known highest position and all 11 brain regions available for analysis (n=385)
CTE: chronic traumatic encephalopathy; NA: not applicable; NFT: neurofibrillary tangle

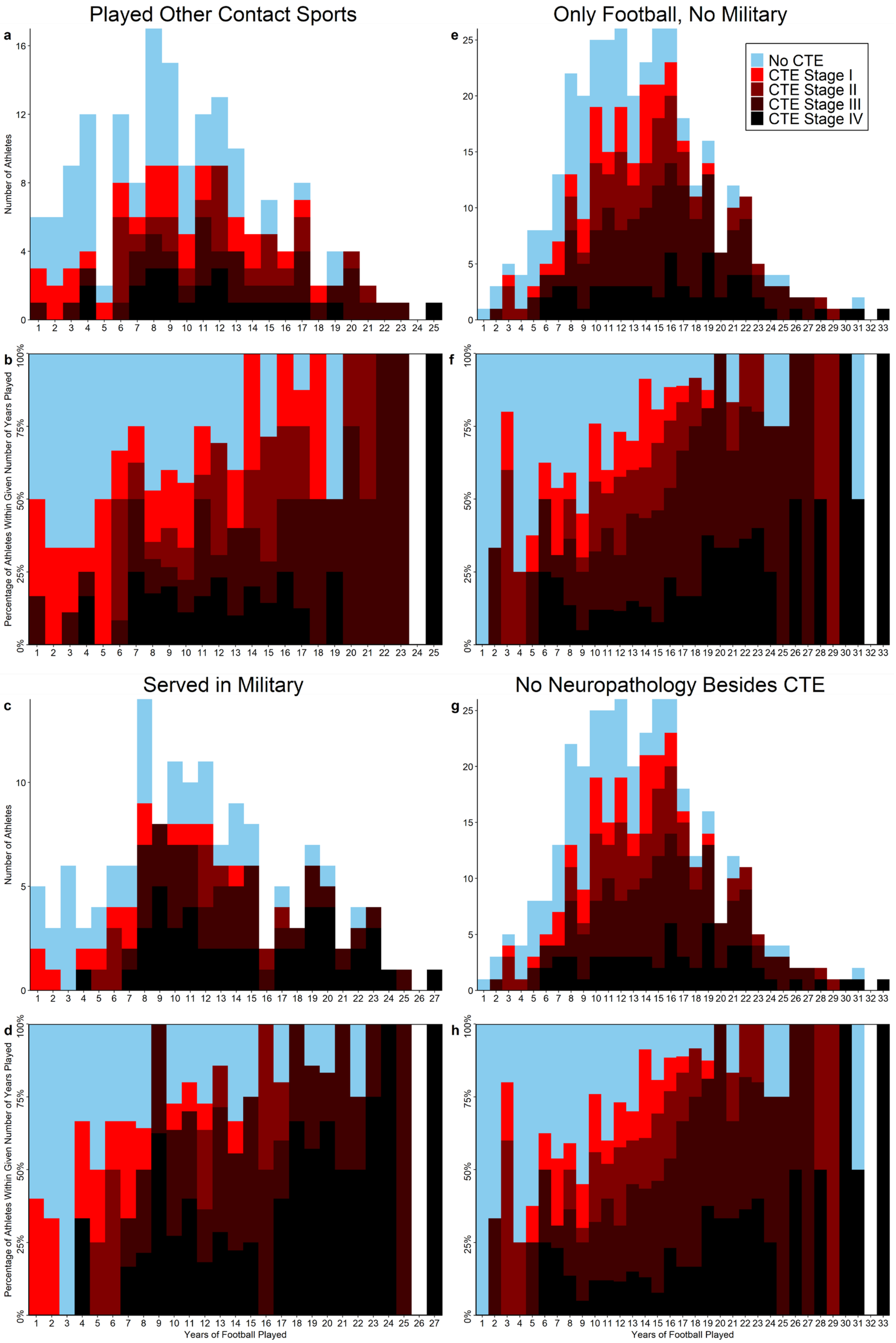

Supplementary Figure 1. Athlete CTE Status by Years of American Football Played for Subgroups. Histograms and percent distributions for subgroups of study sample including football players who also played other contact sports (A and B), football players who also served in the military (C and D), football players who did not play any other contact sports and did not serve in the military (E and F), and football players with no other neuropathology besides CTE (G and H). Source data are provided as a Source Data file. CTE: chronic traumatic encephalopathy

Supplementary Table 5. Association between exposure measures and CTE status (absent versus present) for subgroups

	Odds ratio (95% CI)	p-value	BIC	Cross-validation mean error	AUC	p-value**
No military history and no other contact sport exposure besides football (n=364)						
Concussion number	1.00 (1.00-1.00)	.14				
Position, non-speed vs speed*	1.12 (0.64-2.00)	.68				
Duration of play per year	1.15 (1.09-1.21)	$5.6*10^{-7}$	376.1	0.170	.716	
CHII per 1,000 hits	1.20 (1.10-1.30)	$5.4*10^{-5}$	386.1	0.174	.681	.17
CHII-G per 10,000g	1.22 (1.14-1.31)	$3.7*10^{-9}$	360.6	0.162	.765	$1.0*10^{-3}$
CHII-R per 1,000,000 rad/sec^2	1.26 (1.17-1.36)	$4.5*10^{-9}$	358.9	0.156	.781	$3.6*10^{-5}$
No other neurodegenerative disease besides CTE (n=413)						
Concussion number	1.00 (1.00-1.00)	.35				
Position, non-speed vs speed*	1.59 (0.92-2.7)	0.094				
Duration of play per year	1.16 (1.10-1.21)	$2.6*10^{-9}$	443.2	0.174	.740	
CHII per 1,000 hits	1.18 (1.09-1.27)	$2.5*10^{-5}$	463.9	0.183	.703	.035
CHII-G per 10,000g	1.21 (1.14-1.28)	$6.2*10^{-11}$	431.7	0.168	.776	$5.2*10^{-3}$
CHII-R per 1,000,000 rad/sec^2	1.26 (1.18-1.35)	$2.4*10^{-11}$	426.5	0.166	.789	$3.7*10^{-4}$

Separate logistic regressions were run for each exposure measure due to multicollinearity to determine odds ratios, and p-values. For models with significant exposure measures, BIC and the mean error resulting from a 10-fold cross-validation analysis are reported to determine relative model performance. All models had the outcome of CTE status (absent vs present) and were adjusted for age at death.

*Presented as dichotomous non-speed (offensive and defensive lineman) vs speed (all other positions) for all football players with a single known position

**AUC p-value represents results of bootstrap analysis with 2000 replicates drawn from the sample to determine if there was a true difference between the AUCs for models examining CTE status and duration of play compared to other exposure measures.

AUC: receiver operating characteristics area under curve, BIC: Bayesian information criterion; CHII: cumulative head impact index representing estimated number of head impacts per donor per 1,000 hits; CHII-G: cumulative head impact index representing estimated cumulative g-force experienced by each donor per 10,000g; CHII-R: cumulative head impact index estimated cumulative rotational force experienced by each donor per 1,000,000 rad/sec^2; CTE: chronic traumatic encephalopathy

Supplementary Table 6. Association between exposure measures and CTE severity (mild versus severe) for exposure subgroups

	Odds ratio (95% CI)	p-value	BIC	Cross-validation mean error	AUC	p-value**
No military history and no other contact sport exposure besides football (n=266)						
Concussion number	1.00 (1.00-1.00)	.96				
Position, non-speed vs speed*	1.37 (0.79-2.36)	.26				
Duration of play per year	1.13 (1.06-1.20)	$3.0*10^{-4}$	251.6	0.150	.659	
CHII per 1,000 hits	1.11 (1.11-1.22)	0.022	260.7	0.159	.648	.70
CHII-G per 10,000g	1.21 (1.16-1.31)	$3.6*10^{-6}$	239.8	0.143	.723	$9.4*10^{-6}$
CHII-R per 1,000,000 rad/sec^2	1.21 (1.11-1.32)	$1.2*10^{-5}$	241.8	0.144	.725	$9.7*10^{-4}$
No other neurodegenerative disease besides CTE (n=293)						
Concussion number	1.00 (1.00-1.00)	.41				
Position, non-speed vs speed*	1.61 (0.84-3.09)	.15				
Duration of play per year	1.16 (1.09-1.24)	$3.0*10^{-6}$	264.6	0.138	.739	
CHII per 1,000 hits	1.12 (1.03-1.21)	$9.1*10^{-3}$	283.6	0.152	.703	.031
CHII-G per 10,000g	1.23 (1.14-1.33)	$9.0*10^{-8}$	254.3	0.133	.776	$4.4*10^{-3}$
CHII-R per 1,000,000 rad/sec^2	1.28 (1.17-1.40)	$3.6*10^{-8}$	249.5	0.133	.789	$4.0*10^{-4}$

Separate logistic regressions were run for each exposure measure due to multicollinearity to determine odds ratios and p-values. For models with significant exposure measures, BIC and the mean error resulting from a 10-fold cross-validation analysis are reported to determine relative model performance. All models had the outcome of CTE severity (mild vs severe) and were adjusted for age at death.

*Presented as dichotomous non-speed (offensive and defensive lineman) vs speed (all other positions) for all football players with a single known position

**AUC p-value represents results of bootstrap analysis with 2000 replicates drawn from the sample to determine if there was a true difference between the AUCs for models examining CTE status and duration of play compared to other exposure measures.

AUC: receiver operating characteristics area under curve, BIC: Bayesian information criterion; CHII: cumulative head impact index representing estimated number of head impacts per donor per 1,000 hits; CHII-G: cumulative head impact index representing estimated cumulative g-force experienced by each donor per 10,000g; CHII-R: cumulative head impact index estimated cumulative rotational force experienced by each donor per 1,000,000 rad/sec^2; CTE: chronic traumatic encephalopathy
