# Supplementary material for: Leveraging football accelerometer data to quantify associations between repetitive head impacts and chronic traumatic encephalopathy in males
Source: Nat Commun. 2023 Jun 20;14:3470. doi: 10.1038/s41467-023-39183-0 (PMC10281995; doi:10.1038/s41467-023-39183-0)
Supplement: Supplementary file 2 — Description of Additional Supplementary Files [file 41467_2023_39183_MOESM2_ESM.pdf]

Description of Additional Supplementary Files

Supplementary Code: All code used for the present project, as well as a sample dataset.
