# Supplementary material for: Leveraging football accelerometer data to quantify associations between repetitive head impacts and chronic traumatic encephalopathy in males
Source: Nat Commun. 2023 Jun 20;14:3470. doi: 10.1038/s41467-023-39183-0 (PMC10281995; doi:10.1038/s41467-023-39183-0)
Supplement: Supplementary file 4 — Reporting Summary [file 41467_2023_39183_MOESM4_ESM.pdf]

nature portfolio

Corresponding author(s): Daniel Hamed Daneshvar

Last updated by author(s): May 18, 2023

Reporting Summary

Nature Portfolio wishes to improve the reproducibility of the work that we publish. This form provides structure for consistency and transparency in reporting. For further information on Nature Portfolio policies, see our Editorial Policies and the Editorial Policy Checklist.

Statistics

For all statistical analyses, confirm that the following items are present in the figure legend, table legend, main text, or Methods section.

n/a	Confirmed	
☐	☒	The exact sample size (*n*) for each experimental group/condition, given as a discrete number and unit of measurement
☐	☒	A statement on whether measurements were taken from distinct samples or whether the same sample was measured repeatedly
☐	☒	The statistical test(s) used AND whether they are one- or two-sided<br>*Only common tests should be described solely by name; describe more complex techniques in the Methods section.*
☐	☒	A description of all covariates tested
☐	☒	A description of any assumptions or corrections, such as tests of normality and adjustment for multiple comparisons
☐	☒	A full description of the statistical parameters including central tendency (e.g. means) or other basic estimates (e.g. regression coefficient) AND variation (e.g. standard deviation) or associated estimates of uncertainty (e.g. confidence intervals)
☐	☒	For null hypothesis testing, the test statistic (e.g. *F*, *t*, *r*) with confidence intervals, effect sizes, degrees of freedom and *P* value noted<br>*Give P values as exact values whenever suitable.*
☐	☒	For Bayesian analysis, information on the choice of priors and Markov chain Monte Carlo settings
☐	☒	For hierarchical and complex designs, identification of the appropriate level for tests and full reporting of outcomes
☐	☒	Estimates of effect sizes (e.g. Cohen's *d*, Pearson's *r*), indicating how they were calculated

Our web collection on statistics for biologists contains articles on many of the points above.

Software and code

Policy information about availability of computer code

Data collection	Data was collected and tabulated into REDCap and exported as a csv file
Data analysis	Data analysis was completed in R v4.0.5 or SPSS v.27.0.1.0

For manuscripts utilizing custom algorithms or software that are central to the research but not yet described in published literature, software must be made available to editors and reviewers. We strongly encourage code deposition in a community repository (e.g. GitHub). See the Nature Portfolio guidelines for submitting code & software for further information.

Data

Policy information about availability of data

All manuscripts must include a data availability statement. This statement should provide the following information, where applicable:

- Accession codes, unique identifiers, or web links for publicly available datasets
- A description of any restrictions on data availability
- For clinical datasets or third party data, please ensure that the statement adheres to our policy

The datasets generated during and/or analysed during the current study are not publicly available due to potential ability to identify elite athletes based on exposure data but are available from the corresponding author on reasonable request and with relevant IRB approval.

Human research participants

Policy information about studies involving human research participants and Sex and Gender in Research.

Reporting on sex and gender	Study consisted of football players, predominantly male sport with only men included in analyses. However, repetitive head impact factors could pertain to other exposures, including those more commonly experienced by females as well
Population characteristics	A total of 631 brain donors who played American tackle football were included in the study. Characteristics of all brain donors are presented in Table 1. On average, athletes died at 59.7 years old (SD=20.1) and played 12.5 years of football (SD=5.9). 180 athletes did not have CTE, 163 had low-stage CTE (Stage I or II), and 288 had high-stage CTE (Stage III or IV).
Recruitment	All participants were former football players from the Understanding Neurologic Injury and Traumatic Encephalopathy (UNITE) and Framingham Heart Study (FHS) Brain Banks (Figure 3), which have been previously described.5 Donors to the UNITE Brain Bank must have had a history of repetitive head impact (RHI) exposure through contact and collision sports (CCS), military service, or domestic violence. Recruitment to the UNITE Brain Bank began in 2008 and is ongoing; the present study included donors through 2020. FHS is a prospective surveillance study tracking incident cardiovascular disease, stroke, and dementia. The study was established in 1948, enrolling a representative group from Framingham, Massachusetts and subsequently their children (Gen 2) and grandchildren (Gen 3), as well as ethnically diverse cohorts (Omni 1&2) to better capture changing demographics of Framingham. The FHS Brain Bank contains a voluntary subset of these cohorts, and began recruiting in 1997. Selection bias is inherent to brain donor cohorts
Ethics oversight	Boston University Medical Campus and Bedford VA Hospital IRBs

Note that full information on the approval of the study protocol must also be provided in the manuscript.

Field-specific reporting

Please select the one below that is the best fit for your research. If you are not sure, read the appropriate sections before making your selection.

☒ Life sciences ☐ Behavioural & social sciences ☐ Ecological, evolutionary & environmental sciences

For a reference copy of the document with all sections, see nature.com/documents/nr-reporting-summary-flat.pdf

Life sciences study design

All studies must disclose on these points even when the disclosure is negative.

Sample size	Convenience sample of American football player brain donors. All football playing brain donors through 2020 were included
Data exclusions	For both brain banks, prospective donors were excluded if there was a postmortem interval for donation greater than 72 hours, or if fragments or less than a hemisphere of tissue were received.
Replication	This represents the largest cohort of football playing brain donors published to date. Replication will require new donor cohort
Randomization	No randomization, cohorts determined based on CTE status and severity post-mortem
Blinding	Clinicians and pathologists were blinded until completion of their respective analyses. Because CTE status and severity was determined by pathologists, clinicians collecting clinical data were blinded to group status.

Reporting for specific materials, systems and methods

We require information from authors about some types of materials, experimental systems and methods used in many studies. Here, indicate whether each material, system or method listed is relevant to your study. If you are not sure if a list item applies to your research, read the appropriate section before selecting a response.

Materials & experimental systems

n/a	Involved in the study	
☒	☐	Antibodies
☒	☐	Eukaryotic cell lines
☒	☐	Palaeontology and archaeology
☒	☐	Animals and other organisms
☒	☐	Clinical data
☒	☐	Dual use research of concern

Methods

n/a	Involved in the study	
☒	☐	ChIP-seq
☒	☐	Flow cytometry
☒	☐	MRI-based neuroimaging
